# Pharmacogenomics of Hypertension in Africa: Paving the Way for a Pharmacogenetic-Based Approach for the Treatment of Hypertension in Africans

**DOI:** 10.1155/2023/9919677

**Published:** 2023-05-30

**Authors:** Jonathan N. Katsukunya, Nyarai D. Soko, Jashira Naidoo, Brian Rayner, Dirk Blom, Phumla Sinxadi, Emile R. Chimusa, Michelle Dandara, Kevin Dzobo, Erika Jones, Collet Dandara

**Affiliations:** ^1^Division of Human Genetics, Department of Pathology and Institute of Infectious Disease and Molecular Medicine (IDM), Faculty of Health Sciences, University of Cape Town, Cape Town, South Africa; ^2^UCT/South African Medical Research Council (SAMRC) Platform for Pharmacogenomics Research and Translation Unit, University of Cape Town, Cape Town, South Africa; ^3^Department of Medicine, Division of Nephrology and Hypertension, Groote Schuur Hospital and Faculty of Health Sciences, University of Cape Town, Cape Town, South Africa; ^4^Department of Medicine, Division of Lipidology and Cape Heart Institute, Groote Schuur Hospital and Faculty of Health Sciences, University of Cape Town, Cape Town, South Africa; ^5^Department of Medicine, Division of Clinical Pharmacology, Groote Schuur Hospital and Faculty of Health Sciences, University of Cape Town, Cape Town, South Africa; ^6^Department of Applied Sciences, Faculty of Health and Life Sciences, Northumbria University, Newcastle, Tyne and Wear NE1 8ST, UK; ^7^Medical Research Council-SA Wound Healing Unit, Hair and Skin Research Laboratory, Division of Dermatology, Department of Medicine, Groote Schuur Hospital, Faculty of Health Sciences University of Cape Town, Anzio Road Observatory, Cape Town 7925, South Africa

## Abstract

In Africa, the burden of hypertension has been rising at an alarming rate for the last two decades and is a major cause for cardiovascular disease (CVD) mortality and morbidity. Hypertension is characterised by elevated blood pressure (BP) ≥ 140/90 mmHg. Current hypertension guidelines recommend the use of antihypertensives belonging to the following classes: calcium channel blockers (CCB), angiotensin converting inhibitors (ACEI), angiotensin receptor blockers (ARB), diuretics, *β*-blockers, and mineralocorticoid receptor antagonists (MRAs), to manage hypertension. Still, a considerable number of hypertensives in Africa have their BP uncontrolled due to poor drug response and remain at the risk of CVD events. Genetic factors are a major contributing factor, accounting for 20% to 80% of individual variability in therapy and poor response. Poor response to antihypertensive drug therapy is characterised by elevated BPs and occurrence of adverse drug reactions (ADRs). As a result, there have been numerous studies which have examined the role of genetic variation and its influence on antihypertensive drug response. These studies are predominantly carried out in non-African populations, including Europeans and Asians, with few or no Africans participating. It is important to note that the greatest genetic diversity is observed in African populations as well as the highest prevalence of hypertension. As a result, this warrants a need to focus on how genetic variation affects response to therapeutic interventions used to manage hypertension in African populations. In this paper, we discuss the implications of genetic diversity in *CYP11B2, GRK4, NEDD4L, NPPA, SCNN1B, UMOD, CYP411, WNK, CYP3A4/5, ACE, ADBR1/2, GNB3, NOS3, B2, BEST3, SLC25A31, LRRC15* genes, and chromosome 12q loci on hypertension susceptibility and response to antihypertensive therapy. We show that African populations are poorly explored genetically, and for the few characterised genes, they exhibit qualitative and quantitative differences in the profile of pharmacogene variants when compared to other ethnic groups. We conclude by proposing prioritization of pharmacogenetics research in Africa and possible adoption of pharmacogenetic-guided therapies for hypertension in African patients. Finally, we outline the implications, challenges, and opportunities these studies present for populations of non-European descent.

## 1. Introduction

Hypertension is a major public health concern and is the leading cause of several cardiovascular diseases (CVDs) [1]. Globally, an estimated 1.13 billion individuals have been reported to be hypertensive, and Africa contributes significantly to the increasing global prevalence of hypertension with an estimated prevalence of 27% [2]. The number of hypertensives in Africa is projected to increase by up to 216.8 million by 2030 [3]. Noncommunicable diseases (NCDs) account for approximately 2.8 million deaths in Africa mainly due to ischaemic heart disease (IHD) and stroke for which hypertension is an important risk factor [4] (Table 1). A significant number of individuals are unaware that they are hypertensive and this puts them at increased risk of IHD, stroke, and other CVD events, hence the term “silent killer” [5]. The African Union (AU) also recognises hypertension as one of the major public health challenges in Africa after HIV/AIDS and lobbies towards implementation of effective strategies to screen and manage hypertension including firm stakeholder participation in implementing these policies [6].

The WHO identifies the increasing prevalence of hypertension in Africa to be coupled to low hypertension treatment rates which range from 10% to 21% (Table 2**)** in both males and females [7]. These reflect issues in healthcare systems which fall short in educating and providing therapy which consequently lead to suboptimal blood pressure (BP) control. However, suboptimal BP control in patients receiving medication has been attributed at least in part to poor adherence and persistence in taking drugs throughout long term treatment [8].

Polypharmacy is another challenge, as most hypertensive patients start with one or two antihypertensive drugs with a progressive increase in the number of drugs with increasing time on treatment, age, or comorbidities [9, 10]. According to the REGARDs study, Africans have been shown to have poorer blood pressure (BP) control compared to Europeans [11]. Evidence shows that African patients are susceptible to severe forms of hypertension compared to other ethnicities and require more aggressive treatment, comprising of multiple drugs for adequate BP control [12]. Moreover, other factors have been reported to contribute to poor BP control including diet [13, 14], comorbidities such as diabetes [15], body mass index (BMI) [16], drug-drug interactions [13], and drug-herb interactions. Due to the sociodemographic status of many African countries, most people still rely on traditional herbal medicines to treat hypertension using herbs such as *Lactuca taraxicifolia, Moringa oleifera*, and *Myrothamnus flabellifolius* [17]. Therefore, it is common to get reports of patients taking traditional herbs, while on treatment with conventional drugs [18–20]. Thus, drug-herb interactions could be a contributor to inadequate BP control in African patients. In addition, genetic factors are thought to account for nearly 50% of the variability observed among patients on treatment [21].

Numerous antihypertensive drugs have been approved for use [22–25] and are grouped into six classes; (a) calcium channel blockers (CCB), (b) angiotensin converting inhibitors (ACEI), (c) angiotensin receptor blockers (ARB), (d) diuretics, (e) *β*-blockers, and (f) mineralocorticoid receptor antagonists (MRAs) (Table 3). The current drugs are cleared by *NR1I2, CYP3A4/5, CYP2C9, UGT1A1, UGT2B7, ABCB1, SLC22A1, SLC22A8, SLC22A2*, and *SLC47A1* (more listed in Table 4**)**. Prescribing patterns, profiles of individual drugs, and availability may differ from country to country. The Pan-African Society of Cardiology (PASCAR) and the International Society of Hypertension (ISH) recommend an initial combination of an ACEI/ARB with a CCB or a combination of CCBs with thiazide or thiazide-like diuretics in African patients. Combination therapy has been shown to improve antihypertensive efficacy and results in a 5-fold reduction in BP compared to doubling doses of single agents [30, 31], where BP remains uncontrolled despite the use of 3 or more different classes of drugs including a diuretic, a mineralocorticoid receptor antagonist (MRA) should be added [32, 33], and such situations then reflect resistant hypertension.

Hypertension is a highly complex condition and control of BP involves the interaction of multiple organ systems and several mechanisms of independent or interdependent pathways. These systems or pathways are responsible for controlling peripheral vascular resistance, cardiac output, and regulating sodium and water to maintain intravascular volume, which further explains why multiple drug classes may be required for optimal BP control [34, 35]. These systems and pathways are regulated by many enzymes encoded for by different genes (Table 4) whose polymorphic nature influences antihypertensive drug response further highlighting the significance of pharmacogenetics [36]. This is particularly important in the African populations, where the optimal approach to antihypertensive treatment remains to be defined.

In developed countries, studies have demonstrated the cost-effectiveness of clinical pharmacogenetic testing and its impact in predicting drug response and/or adverse drug reactions [37]. This has led to the implementation of pharmacogenetic testing in clinical practice, a situation that is still a dream in the developing world. Currently, pharmacogenetic testing is being implemented in community pharmacies in the Netherlands, Canada, and Australia [38]. No African country yet has a fully integrated pharmacogenomics platform in routine clinical practice, although African-specific pharmacogenetic variants affecting drugs such as efavirenz [39–42], rosuvastatin [43], imatinib [44], lumefantrine [45], and warfarin [46–48] have been reported.

The paucity of studies in Africans makes the implementation of pharmacogenetics in clinical practice, a distant reality for hypertension among Africans. In Europe, guidelines on therapeutic dose recommendations based on pharmacogenes have been proposed. For example, the Dutch Pharmacogenetic Working Group (DPWG) issued guidelines on therapeutic dose recommendations for metoprolol and carvedilol based on *CYP2D6* genotypes [49] among other drugs. The DPWG regularly reviews these guidelines as more evidence becomes available. Unfortunately for African patients, updates in current recommendations are informed by studies carried out predominantly among European and Asian patients, with no or very few Africans participating. Therefore, the clinical utility of recommendations in Europeans may not be useful among African populations. It is important that many different populations or ethnic groups are recruited into clinical trials because observations from one group may not necessarily inform what will happen in another. For example, based on the four *CYP2D6* phenotype groups, poor metabolizers (PMs), intermediate metabolizers (IMs), normal metabolizers (NMs), and ultrarapid metabolizers (UMs), several studies have reported variable distributions of the *CYP2D6* genotypes across-populations and this impacts on the metabolism and response to many drugs including antihypertensives such as *β*-blockers [50, 51].

African populations are genetically diverse with multiple ethnic groups [52]. The burden of hypertension also differs between ethnic groups. It appears that African populations are susceptible to severe forms of hypertension characterised by enhanced vascular contractility [53], increased salt retention [54–56], and therapeutic resistance [57]. All these factors contribute to higher morbidity, mortality, and significant economic costs in Africa. For example, in South Africa, the overall direct healthcare costs associated with hypertension were estimated to be nearly US $1 billion [58]. The same report states about 8.2 million South Africans are hypertensive equating to approximately US$ 125 per patient. Reports in Kenya, Ethiopia, and Rwanda have estimated average costs per patient of US$ 305 [59], US$ 92 [60], and US$ 25–US$ 80 [61], respectively. Considering the high costs associated with hypertension, one would also expect a significant decline in hypertension-related morbidities and mortalities in Africa. However, in some African studies, there are still reports estimating that nearly 50% of the patients on treatment struggle to achieve good BP responses [62].

In this review, we aim to give an overview on the pharmacogenetic studies of hypertension from population-based studies, clinical trials, case reports, and published controlled studies which have focussed on individuals of African ancestry or descent. This information is needed to improve public health decisions regarding the treatment and management of hypertension on the African continent.

## 2. Literature Review

Literature review was done by accessing PubMed/Medline and Google Scholar databases between January 2022 and February 2023. Searches were limited to articles that could only be accessed in full. Priority was given to articles that focused on the pharmacogenetics of hypertension in the people of African ancestry. The scope of our definition of African Ancestry included Black Africans, Mixed Ancestry Africans, African Americans, and Afro-Caribbeans. The review was narrowed and assessed the pharmacogenetic implications of genes associated with salt-sensitive hypertension, genes associated with the development/risk of hypertension, genes regulating the pharmacokinetics of antihypertensive drugs, genes associated with adverse drug reactions, and genes uncovered by genome-wide association analysis studies (GWAS) in individuals of African descent.

### 2.1. Summary of Literature Search Strategy and Results

Table 4 lists pharmacogenes that have been reported to be involved in the metabolism of antihypertensive drugs and genes associated with hypertension in African populations. Genes implicated in the pharmacogenetics of hypertension were identified in reported African genomic studies (Tables 4 and 5) and are discussed in this review.

### 2.2. Genes Reported to be Associated with Salt-Sensitive Hypertension among Africans

Several studies have pointed out that Africans are more susceptible to salt-sensitive hypertension [63–66]. Africans have been shown to retain an increased amount of sodium and water in the kidney than Europeans [67]. Salt-sensitive hypertension seems predominant among individuals from Southern and Central Africa [55, 68]. Specifically, studies have reported on hypertensive individuals from South Africa [69] and Kenya [70] who were found to have increased circulating sodium levels. The kidney is the main organ that regulates sodium and water balances, thus genes regulating these processes are of pharmacogenetics and pharmacodynamics importance. Thus, genetic variation in genes regulating kidney function plays a role in changes observed in sodium reabsorption [66], and these genes include *CYP11B2, GRK4, NEDD4L, NPPA, SCNN1B*, *UMOD, CYP411,* and *WNK1,* whose variants have been associated with salt-sensitive hypertension in Africans. In addition, variants in these genes have been reported to occur in high frequency among Africans specifically among the Kikuyu/Kalenjin of Kenya and Xhosa/Coloureds (or Mixed Ancestry) of South Africa [12].

There have been some pharmacogenetic studies for most of these genes except for the *UMOD* gene. The *UMOD* gene encodes a urinary protein called uromodulin and among the variants identified by Jones and colleagues [12], no pharmacogenetic studies had explored their functional significance. However, known variants in the *UMOD* gene such as *rs1333226* (g.137485318T>C) and *rs4293393* (c.-39-2490T>C) have been associated with hypertension and CKD among Africans [71, 72]. For example, *rs4293393*, which results in increased levels of uromodulin, influences BP outcomes upon treatment with furosemide. Furosemide is a common loop diuretic which functions by inhibiting sodium reabsorption in the proximal, distal tubules, and the thick ascending loop of Henle [73].

The *SCNN1B* gene located on chromosome 16p12.2 encodes the *β*-subunit of the epithelial sodium channel (ENaC) which regulates sodium ion and water reabsorption. Three missense variants in the *SCNN1B* gene, *rs201279350* (c.617G>A, p.Arg206Gln)*, rs1799980* (c.1325G>T, p.Gly442Val), and *rs149868979* (c.1688G>A, p.Arg563Gln) have been reported [12]. The *R563Q* variant is associated with overactivity of the ENaC and resistant hypertension in African and Mixed Ancestry groups of South Africa and may be associated with BP responses to sodium channel antagonists. Although MRAs such as spironolactone and eplerenone are highly efficacious drugs for resistant hypertension [74, 75], individuals harbouring the *R563Q* variant have poor response, needing the alternative use of amiloride as it directly blocks the ENaC [76]. The study by Jones et al. among Xhosa and Mixed Ancestry patients heterozygous for the *R563Q* variant and a high mean BP of 172/99, administration of 5–10 mg of amiloride as an add-on drug, led to a mean decrease in BP of 36/17 mmHg (*P* < 0.0001) demonstrating the crucial role played by pharmacogenetics in guiding therapy.

The *GRK4* gene, located on chromosome region 4p16.3, encodes the G-protein coupled receptor 4 kinase enzyme which regulates BP through phosphorylation of G-protein coupled receptors (GPCR) [77]. In the kidney, these G-protein coupled receptors in turn control sodium reabsorption [78] which also highlights their role in salt-sensitive hypertension. Of the four *GRK4* variants reported by Jones and colleagues [12], the *GRK4 rs2960306* (c.194G>T, p.Arg65Leu)*, rs1024323* (c.425C>T, p.Ala142Val), and *rs1801058* (c.1457T>C, p.Val486Ala) were previously reported to be associated with altered activity of the protein and hypertension. The *GRK4 rs2960306* and *rs1024323* variants occurring in exon 3 and 5, respectively, are frequent in African populations (MAF > 50%, Table 5) and have been reported to predict BP responses to sodium restriction among Africans [81] and were also found to influence responses to metoprolol [82] and atenolol [83] in African Americans.

According to Bhatnagar et al. (2009), African American male carriers of *GRK4 V142* variant with early hypertensive nephrosclerosis on metoprolol therapy were more likely to reach a mean arterial pressure (MAP) of 107 mmHg as compared to *A142* carriers. Participants with the *A142* variant were also observed to be twice as less likely to reach a MAP target of 107 mmHg if they possessed the *L65* variant. Metoprolol is not widely prescribed in African patients compared to atenolol which has a superior safety profile and a longer half-life; however, the study demonstrated how polymorphic *GRK4* can contribute to BP responses to *β*-blockers. With regard to atenolol, the PEAR (pharmacogenomic evaluation of antihypertensive response) study showed that carriers of the *65L* and *142V* variant alleles presented with poor BP response [83]. Haplotype analysis also revealed that with increasing copies of the *65L-142V* haplotype, worse BP responses were observed.

CYP11B2 gene encodes for the aldosterone synthase enzyme is located on chromosome region 8q24.3, codes for a protein which converts deoxycorticosterone into aldosterone [84]. This conversion is important for the synthesis of the hormone aldosterone which is a key regulator of BP through control of sodium and potassium levels in the body [85]. A variant in the promoter region of *CYP11B2, rs1799998* (c.-344T>C), has been reported in approximately 19% of African Americans, Tunisians, and Egyptians (Table 5). Among Tunisians [86], the *CYP11B2* c.-344T allele has been associated with elevated BP and stroke, while among Egyptians [87], spironolactone efficacy. Specifically, after spironolactone therapy, patients with heart failure and reduced ejection fraction (HfrEF) possessing the TT and TC genotypes had significant improvements in the left ventricular ejection fraction (LVEF), decreased left ventricular end systolic volume (LVESV), and left ventricular end diastolic volume (LVEDV) compared to CC genotypes. The *CYP11B2* c.-344T>C SNP has significant pharmacogenetic implications, and beyond Africa, it has been reported to influence BP responses to other drugs such as benazepril [88], hydrochlorothiazide (HCT) [89], and valsartan [90] which are used by different populations across the world.

*ENaC* is another important gene in hypertension found on chromosome region 12p13. Its expression is regulated by the neural precursor cell expressed developmentally down-regulated 4-like (*NEDD4L*) gene located on chromosome 18q21.31 and encodes E3 ubiquitin ligases [91]. The NEDD4L protein binds to *ENaC* through ubiquitination which leads to channel endocytosis. This suppresses *ENaC* function and inhibits epithelial sodium transport [92, 93]. No studies have been done among Africans on the effects of genetic variation in NEDD4L, despite the crucial role it plays in influencing BP response. According to McDonough et al. [94], *NEDD4L* variants *rs4149601* (c.49-16229G>A), *rs292449* (c.-300G>C), and *rs1008899* (c.-360G>A) seem not to be associated with BP response to HCT among African Americans. However, we still need studies to pronounce the effects of these variants in indigenous African populations.

The *NPPA* gene located on chromosome 1p36.22 encodes for the atrial natriuretic polypeptide (ANP) precursor [95, 96] and a common variant, *NPPA rs5065* (*T2238C,* c.454T>C, p.Ter152Arg) has been reported to be associated with antihypertensive medication effects on cardiovascular disease and BP [97], results which emanated from a huge study assessing about 38,000 patients, of which 35% were Africans on different antihypertensive medication. Overall, the study reported that carriers of the C allele had better CVD outcomes than TT homozygous individuals when comparing chlorthalidone and amlodipine. TT genotype carriers had better outcomes when randomised to amlodipine. In addition, CC genotypes had greater reductions in SBP on chlorthalidone followed by amlodipine, doxazosin, and lisinopril, while those with the TT genotype experienced significantly less SBP reduction [97].

Other genes such as *CYP4A11* and *WNK1* genes also contribute to salt-sensitive hypertension, and variants have been implicated in the pharmacogenetics of antihypertensive drugs in individuals of African Ancestry. The *CYP4A11* gene is located on chromosome 1p33 and encodes the *CYP4A11* enzyme which converts arachidonic acid to 20-hydroxyeicosatetraenoic acid (20-HETE). The product, 20-HETE, in turn induces natriuresis through inhibition of the *ENaC* [98]. Given the significance of the *CYP4A11* gene in regulating the *ENaC*, variants that reduce the activity of the enzyme may promote hypertension due to increased sodium/water retention and some variants have been reported to influence BP responses to drugs acting on the *ENaC* pathway. In a study by Laffer et al., the *CYP4A11 rs3890011* (g.46933071G>C) (Table 5), was reported to influence BP responses upon spironolactone or amiloride treatment among African American hypertensives [99].

The *WNK1* gene located on chromosome 12p13.33, having 150 kb, encodes a protein responsible for sodium ion regulation in the kidney [100, 101]. Reduced function of the protein may be implicated in the pathogenesis of hypertension. Three SNPs, *rs2107614* (c.760-19729T>C), *rs2277869* (c.7400-103T>C), and *rs1159744* (c.933-1363G>C), with frequencies ranging from 18% to 47% in Africans (Table 5), encode a lysine deficient kinase. These SNPs have been linked to ambulatory BP responses to hydrochlorothiazide (HCT) among African Americans [102]. In this study, the *rs1159744*G>C was associated with the greatest decrease in ambulatory BP responses to HCT and was also associated with significant differences in urinary potassium excretion. A study by Masilela et al., among Nguni South African patients on HCT, reported an association of the *WNK1 rs2107614* SNP with uncontrolled hypertension (>140/90 mmHg) [103].

The *CYP3A5* gene has 14 exons, ∼33 kb long, and is located on chromosome *7q22*.1. *CYP3A5* is an important gene whose association with hypertension has been established in several studies and has also been linked to salt sensitivity. The *CYP3A5* gene is expressed in the liver and extrahepatic tissues such as the small intestines and kidney [104]. In the kidney, CYP3A5 is responsible for the conversion of cortisol to 6-*β*-hydroxycortisol which enhances sodium and water retention, thereby contributing to hypertension. The levels of the *CYP3A5* enzyme in the kidney have been reported to be high in individuals possessing *CYP3A5∗1* (*rs15524,* g.36708T>C) as compared to *CYP3A5∗3* (*rs776746*, g.99270539C>T) carriers [105]. This highlights that carriers of the *CYP3A5∗1* allele are at increased risk of developing salt-sensitive hypertension, and across ethnic groups, there are variable frequency distributions of the *CYP3A5∗1* allele. Interestingly, 70% of individuals of African descent [106] possess this allele compared to 7% among Europeans [107], which also may explain why Africans are more susceptible to salt-sensitive hypertension. The *CYP3A5∗3* variant is also a common variant that results in a *CYP3A5* nonfunctional protein. Its distribution among African populations is variable; however, according to [104], high frequencies are observed with increasing distance from the equator which explains why the frequencies are higher in Europeans and Asians as compared to Africans (Table 5). Still, the frequencies in Africans are significant (>10%) and this means that this variant significantly affects drugs metabolised by *CYP3A5* such as amlodipine. Other commonly studied variants, such as the *CYP3A5∗6* (*rs10264272,* 14690G>A) and *∗7* (*rs41303343,* 27131–27132insT), are also common among Africans and have been implicated in the production of a nonfunctional protein which means they may have an impact in the disposition of amlodipine [108]. However, a few pharmacogenetic studies in Africans have reported on role of *CYP3A5* variants on therapeutic response to antihypertensive drugs.

### 2.3. Genes That Have Been Shown to be Associated with Hypertension in Africans

A systematic review by Mabhida et al. [29] outlines 53 genes that have been previously studied in African populations and have been associated with hypertension and/or risk. Among these are pharmacogenetic studies on *ACE, ADBR2, GNB3,* and *NOS3* genes. The *ACE* gene, which is located on chromosome 17q23.3, encodes the angiotensin converting enzyme which is the primary target of *ACE* inhibitors such as enalapril. An insertion/deletion (I/*D*) variant, *rs1799752,* in the intron of the *ACE* gene has been linked to hypertension in studies among Tunisians, Egyptians, and black South Africans and has been shown to contribute to *ACE*-inhibitor-induced adverse reactions.

The *ADBR2* gene found on chromosome 5q32 encodes the beta-2-adrenergic receptor. The beta-2-adrenergic receptors are predominant in smooth muscle cells and also in cardiac muscles cells and vascular endothelium. They bind to catecholamines and transmit signals through G-proteins and cyclic adenosine monophosphate (cAMP) which acts as a second messenger [109]. The *ADBR2 rs2400707* SNP has been reported to influence BP responses to HCT among individuals of African Ancestry. In a study by Turner et al. [102], *ADBR2 rs2400707G>A* was reported to cause a significant reduction in ambulatory SBP, but not DBP, among the study participants who included Africans. The same SNP was also studied by Masilela et al. among South African Nguni tribes [103]. In this study, they show that A-allele carriers exhibited better BP control and were less likely to develop uncontrolled hypertension.

Guerra et al. [110] also investigated the influence of genetic variation in the *ADBR2* gene together with the beta-1-adrenergic receptor (*ADBR1*) gene which is on chromosome 10q25.3, on differential survival in heart failure patients on beta-blockers. Here, they showed that *ADBR2 rs1042714* (c.79G>T, p.Glu27Gln) and *ADRB1 rs1801253* (c.1165G>C, p.Gly389Arg) SNPs influenced survival in heart failure patients taking a high beta-blocker dose, highlighting how pharmacogenetic markers may have a potential use in the creation of dosing algorithms in clinical practice.

The same study [110] investigated the influence of the *rs5443* (c.825C>T) SNP in the *GNB3* gene which encodes the guanine nucleotide-binding protein *β*-polypeptide 3, but did not report on the effects of this SNP. However, in other African studies, the SNP was identified in Tunisian populations [111] and has been linked to BP responses to thiazide diuretics in individuals of African descent [112]. In this study, Turner et al. observed that the *T* allele played a role in the reduction of SBP and DBP.

The SNPs *rs1799983* (c.894T>G, p.Asp298Glu)*, rs2070744* (c.-149 + 1691C>T), and *rs61722009* in the *NOS3* gene have been associated with the risk of hypertension among Algerians [113] and Tunisians [114]. According to a study by Masilela et al., the *rs2070744* SNP was associated with BP response to enalapril among Black South Africans [115]. However, the influence of this SNP was only significant in the presence of the *rs699947* and *rs495828* SNPs in the *VEGFA* and *ABO* genes, respectively. Specifically, the combination of the GG, CT, and CC genotypes for the *rs495828*, *rs2070744,* and *rs699947* SNPs, respectively, was associated with uncontrolled hypertension.

### 2.4. Genes Regulating the Pharmacokinetics of Antihypertensive Drugs in Africans

Several studies have identified SNPs in genes that affect the pharmacokinetics of drugs used in the treatment of hypertension which include *ABCB1*, *CES1, CYP2C9*, *CYP2D6*, *CYP3A4, CYP3A5,* and *SLCO1B1. CYP2D6* nonfunctional and reduced function variants have been profiled and identified among Africans [48, 116]. Among South Africans, Dandara et al. identified SNPs *rs1045642, rs1128503, rs2235048,* and *rs3213619* in the *ABCB1* gene; *rs1799853* in the *CYP2C9* gene; *rs2740574* in the *CYP3A4;* and *rs776746* in the *CYP3A5* gene [117]. Out of all these SNPs, only the *rs2740574* and *rs776746* SNP have been reported to be associated with BP response to calcium channel blockers in individuals of African descent. According to Bhatnagar et al., BP response to amlodipine was associated with *CYP3A4* genotypes in a gender-specific manner among African Americans. In this study, female A-allele carriers for the *rs2740574* SNP had triple the chances of achieving a MAP target of 107 mm Hg, while C allele carriers for the *rs35599367* SNP were associated with poor response to amlodipine irrespective of gender [118].

### 2.5. Genes Associated with Adverse Drug Reactions (ADR) in Africans

Poor response to antihypertensive drugs may be characterised either by failure to reach BP targets, or by the occurrence of adverse effects, which have been reported to occur in 25% of hypertensive patients [119, 120]. Multiple factors are responsible for the occurrence of adverse events including genetic predisposition. In African patients, a Nigerian study [119] profiled the occurrence of adverse events in hypertensive patients and reports that diuretics, *ACE* inhibitors, and CCBs accounted for 27.9%, 26.8%, and 26.8% of all adverse reactions, respectively. Although this is not representative of the profile of adverse events to antihypertensive drug use in the whole of Africa, there are reports that *ACE* inhibitor-induced adverse events are frequent in individuals of African descent [121]. The Pan-African Society of Cardiology (PASCAR) also mentions that the use of *ACE* inhibitors in African patients is associated with the risk of adverse events and recommends alternatives such as angiotensin receptor blockers (ARBs). However, the cost-effectiveness of *ACE* inhibitors over ARBs makes them widely used in African countries especially in hypertensive patients not experiencing *ACE*-induced adverse events [32].

The primary target of ACE inhibitors is the angiotensin converting enzyme that converts angiotensin I (Ang I) to angiotensin II (Ang II) which causes vasoconstriction, increased sodium, and fluid retention [122]. Furthermore, the angiotensin-converting enzyme also breaks down bradykinin. Thus, inhibition of the angiotensin-converting enzyme by *ACE* inhibitors results in reduced levels of Ang II, which is also coupled to increased levels of bradykinin, a vasodilator which exerts its effects through binding to the B2 receptor. The B2 receptor is coupled to *G*-proteins and the effects of bradykinin are through the GPCR signalling pathway [123, 124]. This demonstrates the crucial role played by the angiotensin converting enzyme and the B2 receptor in regulating BP. Thus, genetic variation in genes encoding these proteins has been the subject of a number of pharmacogenetic studies which have reported associations of genetic variants with *ACE* inhibitor-induced adverse events such as angioedema or cough and also BP response [125].

Our literature search identified one study among Africans [126] which reported on the angiotensin-converting enzyme gene insertion/deletion (*I*/*D*) polymorphism, while establishing an association of the 9/+9 insertion/deletion polymorphism in the *B2* gene with *ACE*-induced angioedema and cough. Briefly, Black African and Mixed Ancestry participants in the study who were carriers of the −9/+9 genotype had *ACE*-induced angioedema or cough and this association remained true upon further analysis in the dominant model, which implicated the *B2* −9 allele to the observed associations. It is the accumulation of bradykinin that results in angioedema and cough which accompanies *ACE* inhibitor use [126].

### 2.6. Genes and Loci Identified in GWAS Specific to African Descent Populations

The advent of newer and powerful genomic sequencing technologies has enabled researchers to perform more in-depth genomic studies such as genome-wide-association studies (GWAS) which search for small genetic variations (or SNPs) across the genome with wide coverage. This has been important in the study of complex conditions such as hypertension and GWAS have further uncovered novel genes or loci involved in BP regulation, progression of hypertension, and drug response [127]. Among these genes, the genes *SLC25A31, LRRC15* [128], and loci such as the chromosome 12q [129] have been uncovered by GWAS and have been reported to influence BP responses to antihypertensive drugs in Africans.

A GWAS by Gong et al. [128] including African Americans, on atenolol and metoprolol therapy, identified SNPs in the *SLC25A31* and *LRRC15* genes associated with BP response. The *SLC25A31* gene located on chromosome 4q28.1 encodes an ATP/ADP translocase that mediates ATP/ADP exchange in the mitochondrial matrix. In this study, an intronic variant *SLC25A31 rs201279313* (c.232 + 5111_232 + 5113del), which is a deletion of TTA, was associated with DBP response to atenolol, metoprolol, and atenolol add-on therapy. Individuals possessing the TTA deletion had significant DBP reductions on atenolol, metoprolol, and atenolol add-on therapy, respectively, compared to the wild type genotype carriers. The *LRRC15* gene is located on chromosomes 3q29 and encodes a 15-leucine-rich repeat containing membrane protein. In the same study, Gong et al. identified the intronic variant *LRRC15 rs11313667* (c.16-387del) which has a MAF of 18% in Africans. This SNP was associated with BP reductions to *β*-blocker therapy, with significant BP reductions observed for SBPs among individuals on atenolol, metoprolol, and atenolol add-on therapy [128].

Turner et al. [129] also conducted a GWAS across 22 autosomes and identified the chromosome 12q15 loci harbouring SNPs *rs317689*, *rs315135,* and *rs7297610* located near the *LYZ, YEATS4,* and *FRS2* genes. This locus was found to be associated with DBP responses to HCT. Out of the haplotypes constructed from the *rs317689, rs315135,* and *rs7297610* SNPs, the A-T-C haplotype was more frequent in African good responders, while A-C-T and A-T-T haplotypes were more frequent in African poor responders, respectively. This association appeared to be more common with the *rs7297610* SNP. In this study, DBP response was mostly driven by variation in the *YEATS4* gene, and upon further analysis, the *YEATS4* gene remained more significantly associated with DBP response to HCT as compared to *LYZ* or *FRS2* genes [129].

The association of the chromosome 12q15 locus with BP response was further confirmed by Duarte et al. [130] in a study including African Americans on HCT and atenolol. In this study, the *rs7297610* SNP was significantly associated with BP response to HCT among African Americans. Carriers of the CC genotype had greater SBP and DBP responses to HCT compared to T-allele carriers. In addition, SNPs, *rs10784780,* and *rs10878983* had significant influences on BP responses to HCT and appeared to be in linkage disequilibrium with each other. Gene expression analyses also showed that CC homozygotes had higher YEATS4 levels which decreased by 15% after HCT treatment further demonstrating the pharmacogenomic effect of the chromosome 12q locus. Masilela et al. [103] also reported on the *rs7297610* SNP uncovered by GWAS. In this study, the *T* allele was found to be associated with uncontrolled hypertension among Swati and Zulu participants, while C allele carriers were less likely to have uncontrolled hypertension further confirming previous findings by Turner et al. [129, 130].

Resequencing of the chromosomal region 12q15 by Singh et al. [131] identified a novel SNP *rs61747221* (c.1148C>T, p.Pro383Leu) in the *BEST3* gene among the study participants which included African Americans. The *rs61747221* SNP was significantly associated with BP responses to HCT. All analyses and subsequent analyses included African Americans and identified carriers of the A-allele (AA + AG) for the *rs61747221* SNP to be associated with BP responses to HCT as compared to GG homozygotes.

## 3. Discussion and Future Prospects

Pharmacogenetic studies of antihypertensive drugs have explored the influence of genetics on drug response in Europeans and Asians as evident in reviews by Rysz et al. [21, 125, 132]. Our review focussed on presenting an African perspective on the pharmacogenetics of hypertension through assessing the pharmacogenetic implications of genes associated with salt-sensitive hypertension, genes associated with the development/risk of hypertension, genes regulating the pharmacokinetics of antihypertensive drugs, genes associated with adverse drug reactions, and genes uncovered by genome-wide association analysis studies (GWAS) in individuals of African descent. Overall, the synthesis of this review shows (i) a lack of genomics and pharmacogenetic studies focussing on hypertension in native African populations, (ii) that genes with pharmacogenetic implications highlighted in this review have variants which exist in significant frequencies (MAF > 5%) in African populations (Table 5), and (iii) that African populations may present with genetic variants that may not be reported in other populations, yet these variants play significant roles in determining therapeutic drug response.

Africans have been underrepresented in most genomics studies of hypertension. There is therefore little evidence to inform how Africans are likely to respond to most antihypertensive drugs. Africans may be more susceptible to salt-sensitive hypertension or resistant hypertension. The pharmacogenetic implications of genes associated with salt sensitivity in this review have been widely reported for African Americans but little is known in native African populations. Of the genes explored in Egyptian and South African pharmacogenetic studies, only the *R563Q* (c.1688G>A, p.Arg563Gln) variant in the *SCNN1B* gene has been validated in clinical studies. Currently, as part of routine clinical practise, genotyping for the *R563Q* variant in the *β*-subunit of the epithelial sodium channel (*ENaC*) is seen guiding BP therapy in South Africa. This demonstrates how genetics has the ability to guide BP therapies as carriers of this variant tend to respond better to amiloride treatment.

Polymorphisms in genes regulating pathways involved in hypertension have been linked to the development of hypertension and/or adverse CVD outcomes in African populations [29]. These genes are also important in predicting drug response; however, available pharmacogenetic studies in populations of African Ancestry have focussed on *ADBR2, GNB3,* and *NOS3* genes although a substantial number of genes have been reported to be associated with hypertension. The few African studies exploring the influence of genetic variation in the *ADBR2* and *NOS3* genes on HCT and enalapril have reported on significant associations with uncontrolled hypertension, and these need to be replicated in other ethnic groups.

Pharmacokinetics of a drug governs its absorption, distribution, metabolism, elimination, and transport (ADMET) [133]. Most antihypertensive drugs do not undergo first pass metabolism [134] and the few that do such as CCBs, *β*-blockers, some ACEI, and some ARBs are metabolised by enzymes and proteins encoded for by *ABCB1, CES1, CYP2C9, CYP2D6, CYP3A4, CYP3A5,* and *SLCO1B1* genes. No pharmacogenetic studies were identified in native African populations assessing the implications of genetic variants in these genes on BP response. However, among African American populations, genetic variation in *CYP3A4* and *CYP3A5* genes has been linked to BP responses to amlodipine [118]. These pharmacokinetic genes are highly polymorphic, and some polymorphisms may encode reduced or nonfunctional enzymes or transporters. This leads to altered BP response and/or accumulation of the parent drug or its metabolites contributing to adverse drug reactions (ADR).

Antihypertensive ADRs may be mild, and some may be potentially life threatening such as *ACE* inhibitor-induced angioedema [126]. According to Leporini et al. [135], ADRs are a key factor contributing to poor medication adherence and contribute to poor control of many chronic diseases including hypertension [136]. In addition to genetic variation in pharmacokinetic genes, variation in pharmacodynamics genes also contribute to ADRs. Currently, a few studies have examined the impact of these genes and the incidence of ADRs in African populations.

Genome-wide association studies (GWAS) have been of critical importance in pharmacogenetic studies of hypertension in European and Asian populations. This approach has facilitated the discovery of novel variants associated with BP response to antihypertensive drugs. In our search, we could not identify any GWAS conducted in Africa. This limits the equitability in the development of hypertension association studies, and polygenic risk scores, their applications to understand hypertension aetiology, and their evaluation for the clinical utility to advance predictive and preventative medicine. However, some GWAS conducted have included African Americans and have uncovered loci and genes which have an influence on BP responses to HCT and *β*-blockers [128–130]. There still remains a need to replicate these findings from GWAS especially in African populations living in Africa.

On the other hand, current GWAS, polygenic risk scores models, and pharmacogenetic studies have not been comprehensively evaluated in large samples of admixed and African-descent populations and face critical challenges in cross-population transferability. We hypothesize that understanding and then appropriately modelling the different aspects of African genetic architecture have the potential to achieve unbiased and powerful estimates of genetic risk across African, multiethnic, and admixed populations. There is therefore an urgent need to optimize GWAS, polygenic risk scores models, and pharmacogenetic studies for analysing admixed and African-descent genetic epidemiological data given the rich complexity and diversity of their genetic characteristics and the emerging proliferation of next generation sequencing technologies.

Due to lack of data, extrapolations on Africans have been done from African American populations in most pharmacogenetic studies, which is not accurate due to the huge genetic diversity of African populations. We call upon for concerted efforts to carry out massive genomics studies on African populations including pharmacogenomics as it is listed as one of the five priorities for genomics in Africa [137].

### 3.1. Presenting a Case for Africa and Concluding Remarks

Cardiovascular diseases have been the topmost leading cause of death globally. Africa has not been spared in mortalities due to cardiovascular diseases and deaths have been seen to be rising in past years with hypertension being the leading risk factor. This has called for the need for better, more efficient, and cost-effective solutions to manage hypertension and pharmacogenetics has a potential of integrating all these solutions. However, the mammoth challenge is that there are currently limited pharmacogenetic studies in African populations focussing on hypertension and therefore the potential of pharmacogenetics cannot be fully harnessed as studies are few to bridge the gap between clinical implementations of pharmacogenetics [137, 138].

As we are in an era of evidence based medicine, where a patient's diagnosis, prognosis, and even therapy are dependent on findings from the best scientific evidence from clinical research [139], more pharmacogenetic studies on hypertension in African populations could actually be a precursor towards a pharmacogenetic-based approach for the treatment of hypertension. However, there is a need to gather data on the prevalence of hypertension and its subtypes as an initial step, including treatment strategies in different African countries. This will provide a basis for future pharmacogenetic studies and assist in identifying which countries where hypertension pharmacogenetic studies should be prioritised, further assisting stake-holder decision making. There are already wide differences in the prevalence of hypertension between African countries with some bearing a high burden, thus public health sector priorities differ by country [140].

Pharmacogenetics presents as an attractive approach for optimum management of hypertension in Africa. Most hypertensive individuals, especially Africans, are rarely on one drug but require multiple drugs to keep their BP controlled. This most often involves a number of trial and error approaches until optimal drug combinations are found. Pharmacogenetics has the ability to assist in “tailor-making” drug/doses [141] and has the potential to contribute to better outcomes including reduced occurrence adverse drug events, fewer hospital visits, reduced number of drugs per patient, and better adherence to medication, consequently reducing cardiovascular morbidities and mortalities in the context of hypertension.

In other parts of the world, where pharmacogenetic implementation studies have been carried out, clinicians have reported improved clinical outcomes in patients who had undergone pharmacogenetic testing prior [38, 142–144]. As a result, dosing algorithms for drugs/drug classes such as clopidogrel, tamoxifen, and warfarin/statins [145–147], have now been developed for Europeans and Asians and these are now gaining momentum in Africa, with promising potential for implementation, as more data are being generated for native and admixed African populations [43, 46, 47, 138, 148, 149]. For hypertension, only metoprolol has dosing guidelines based on *CYP2D6* genotypes proposed by the DPWG. However, the recommendations have limited applications for African populations [49]. Furthermore, genotyping, for the *R563Q* (c.1688G>A, p.Arg563Gln) variant in the *SCNN1B* gene, is seen guiding amiloride therapy in South Africa. However, there is still room for expanding the spectrum of genes and variants that can be used to guide antihypertensive drug therapy since hypertension is a polygenic condition.

It is a well-known fact that Africa is also heavily burdened by infectious diseases such as HIV/AIDs and schistosomiasis. This has, in past years, stimulated stakeholder interest and funds have been mobilised to support research in these areas. Researchers across Africa have actively participated in pharmacogenetic research in this area with emanated dosing guidelines for efavirenz and praziquantel [150–152], which have found their way into the clinic. The same could also be realised for hypertension, where genetics could inform therapy seeing that it has now been “dubbed” a major public health concern in Africa after HIV/AIDs [3, 6]. To further strengthen our case, pharmacogenetics is a proven cost-cutting solution to medication costs. A study by Javis et al. reports a US $7000 reduction per patient in medical costs for patients who underwent pre-emptive pharmacogenetic testing, an observation also supported by a recent study by Swen et al. [153, 154]. Given that a pharmacogenetic test is for life, this would be a huge cut down on treatment costs, especially for low-middle income African countries burdened by hypertension.

Thus, there needs to be active participation towards the consolidation of pre-emptive pharmacogenetic testing and pharmacogenetic-guided dosing algorithms into clinical practise in Africa, especially for hypertension. This may include mobilisation of systems ensuring access to facilities and infrastructure that offer pharmacogenetic-based testing or treatment as a standard part of care, pre- and postpharmacogenetic counselling, pharmacovigilance, and training for healthcare providers [143]. One of the United Nations (UN) sustainable development goals is a one-third reduction in deaths due to NCDs by the year 2030. Therefore, we call on policy makers in Africa and international partners to provide the necessary environment (funding and easy of movement) to enable researchers to engage and foster collaborative activities that will lead to the decoding of the genomic variation among African populations which affects disease susceptibility and therapeutic drug responses.

Hypertension is a modifiable risk factor for CVDs and can be manageable through effective treatment. Thus, more pharmacogenetic research in this area can help unravel crucial pharmacogenes and variants, with potential for clinical translation that may improve the quality of life in African patients. This may seem as a daunting task; however, the vision has already been conceptualised by the African Pharmacogenomics Consortium (APC) [155], which has proposed strategies towards the implementation and consolidation of pharmacogenetics in Africa which are highly relevant for noncommunicable diseases such as hypertension.

## Figures and Tables

**Table 1:** The top five causes of death in Africa recorded in 2019 by the World Health Organisation (WHO).

Cause of death	Communicable/Noncommunicable	Number of deaths	Percentage (%) of total deaths in 2019
Lower respiratory tract infections	Communicable	774 252	9.9
Diarrhoea	Communicable	496 278	6.4
HIV/AIDS	Communicable	434 543	5.6
Ischaemic heart disease and stroke	Noncommunicable	429 179	5.5^*∗∗∗*^
Parasitic diseases	Communicable	388 229	5.0

^*∗∗∗*^Hypertension is the leading risk factor for ischaemic heart disease and stroke. Noncommunicable diseases rank fourth and are potentiated by hypertension.

**Table 2:** List of top African countries with low hypertension treatment rates as reported by the World Health Organisation in 2021 by gender.

Country	Treatment rate (%)
*Women*	
Rwanda	11
Niger	15
Ethiopia	15
Tanzania	17
Madagascar	19
Mozambique	19
Kenya	21

*Men*	
Rwanda	10
Kenya	10
Mozambique	10
Niger	12
Madagascar	13
Uganda	13
Togo	14
Burkina Faso	14

Treatment rate (%) expressed as the percentage of all women or men with hypertension.

**Table 3:** List of FDA-approved antihypertensive drugs.

Class of antihypertensive drug	Names of drugs in the respective classes
Calcium channel blockers (CCBs)	**Amlodipine, nifedipine**, nilsodipine, **diltiazem**, felodipine, isradipine, nicardipine, **verapamil**, and nimodipine
Angiotensin-converting inhibitors (ACEI)	Captopril, **enalapril**, **lisinopril**, perindopril, quinapril, ramipril, trandolapril, **benazepril**, fosinopril, and moxipril
Angiotensin receptor blockers (ARBs)	Eprosartan, **candesartan**, **losartan**, **valsartan**, irbesartan, telmisartan, azilsartan, and olmesartan
Diuretics	**Hydrochlorothiazide**, chorthalidone, chlorothiazide, metolazone, ethacrynic acid, indapamide, **furosemide**, bumetanide, torsemide, **amiloride**, and triamterene
*α* and *β*-blockers	**Atenolol**, bisoprolol, metoprolol, propranolol, **carvedilol**, **doxazosin**, prazosin, and terazosin
Mineralocorticoid receptor antagonists (MRA)	Eplerenone, **spironolactone**, and finerenone
Direct vasodilators	**Hydralazine** and **minoxidil**
Peripherally acting adrenergic antagonists	Reserpine

Source: [26]. Drugs used among African patients (**in bold)** obtained from studies from different African countries [22–25] and from unpublished data collected and managed using REDCap electronic data capture tools hosted at the Hypertension Clinic Groote Schuur Hospital, Cape Town, South Africa [27, 28]. The drugs highlighted in bold have been reported to be used among African patients.

**Table 4:** Genes that have been studied for their effect on hypertension susceptibility or response (pharmacogenes).

Pharmacogenes		Susceptibility genes		
ABCB1	**CYP3A4**	ADD1	GNAS-EDN3	MTHFR
**ACE**	**CYP3A5**	AGT	**GNB3**	**NOS3**
**ADBR2**	NAT2	APOA5	GOSR2	**NPPA**
**ADRB1**	NR3C2	ATP2B1		**NEDD4L**
AGTR1	**SCNN1**	**B2**	GPR83	PLCE1
CACNA1C	SLC12A1	BAG6	**GRK4**	PLEKHA7
CACNA1D	SLC12A3	CDKAL1	HFE	PR3
CACNB2	SLCO1B1	CEP83	HSD3B1	SH2B3
CES1	UGT	CHIC2	IGF2BP2	SLC39A8
CYP1A1		CLCNKB1	IGFBP3	SLC4A7
CYP1A2		CNNM2	JAG1	STK39
CYP2C8		CPS1	KCNJ1	SUB1
CYP2C9		**CYP11B2**	LEP	TBX5
CYP2D6		**CYP411**	LUC7L2	TH
CYP2E1		EBF1	MECOM	**WNK1**
		FGF5	MOV10	**WNK4**

Genes listed under pharmacogenes are involved in the metabolism of antihypertensive drugs. Genes listed under susceptibility genes have been associated with hypertension in African populations [29]. Highlighted in bold are genes with pharmacogenetic studies done in individuals of African descent.

**Table 5:** The distribution of minor allele frequencies (MAF) in genes that have been studied among African populations.

Gene	Polymorphism	Allele	African				European	Asian
			Bantu (Southern Africa)	Luhya (Kenya)	Yoruba (Nigeria)	African American		
CYP11B2	*c.-344T>C (rs1799998)*	C	—	0.20	0.14	0.19	0.49	0.39

GRK4	*c.194G>T, p.Arg65Leu (rs2960306)*	T	0.50	0.60	0.55	0.45	0.38	0.11
	*c.425C>T, p.Ala142Val (rs1024323)*	T	0.50	0.64	0.67	0.58	0.40	0.19
	*c.1457T>C, p.Val486Ala (rs1801058)*	T	—	0.06	0.09	0.16	0.43	0.46

NEDD4L	*c.49-16229G>A (rs4149601)*	A	0.69	0.50	0.38	0.34	0.35	0.16
	*c.-300G>C (rs292449)*	G	—	0.47	0.45	0.49	0.66	0.13
	*c.-360G>A (rs1008899)*	A	0.06	0.08	0.04	0.11	0.25	0.45
NPPA	*c.454T>C, p.Ter152Arg (rs5065)*	G	0.56	0.31	0.43	0.40	0.12	0.01

SCNN1B	*c.1688G>A, p.Arg563Gln (rs149868979)*	G	0.06	0.00	0.00	0.00	0.00	0.00

CYP411	*g.46933071G>C (rs3890011)*	C	—	0.32	0.36	0.44	0.22	0.41

WNK1	*c.760-19729T>C (rs2107614)*	C	—	0.53	0.39	0.47	0.68	0.84
	*c.7400-103T>C (rs2277869)*	C	—	0.18	0.13	0.16	0.14	0.16
	*c.933-1363G>C (rs1159744)*	C	—	0.23	0.28	0.25	0.26	0.10

ADBR1	*c.1165G>C, p.Gly389Arg (rs1801253)*	G	—	0.31	0.48	0.41	0.32	0.35
ADBR2	*g.148825489A>G (rs2400707)*	A	0.50	0.57	0.43	0.50	0.59	0.75
	*c.79G>C, T p.Glu27Gln (rs1042714)*	G	—	0.21	0.12	0.20	0.41	0.14

GNB3	*c.825C>T (rs5443)*	T	—	—	—	0.76	0.31	0.54

NOS3	*c.894T>G, p.Asp298Glu (rs1799983)*	T	0.08	0.04	0.06	0.11	0.34	0.15
	*c.-149+1691C>T (rs2070744)*	C	0.10	0.14	0.12	0.16	0.44	0.10

CYP3A4	*c.-392G>A (rs2740574)*	C	—	0.17	0.24	0.45	0.03	0.00
	*g.99768693G>A (rs35599367)*	A	0.03	0.00	0.00	0.00	0.05	0.00

CYP3A5	*g.99672916T>C (rs776746)*	C	0.19	0.12	0.17	0.31	0.94	0.71

LRRC15	*g.194361436del (rs11313667)*	CCC	—	0.76	0.85	0.76	0.47	0.70

Chromosome 12q	*g.69430244C>T (rs7297610)*	T	0.13	0.31	0.37	0.30	0.07	0.00
	*g.69333410G>A (rs317689)*	G	0.13	0.14	0.08	0.14	0.28	0.26
	*g.69369207A>G (rs315135)*	G	0.13	0.22	0.14	0.12	0.05	0.00

BEST3	*g.a69643740G>A, p.Pro383Leu (rs61747221)*	A	—	0.14	0.15	0.16	0.09	0.26

^*∗*^Frequencies obtained from the allele frequency database for Bantu populations, and 1,000 genomes database for other African, European, and Asian populations [79, 80].

## Data Availability

No data were used to support this study.
